# Clinical Electro-Therapeutics, Medical and Surgical

**Published:** 1873-11

**Authors:** 


*Clinical Electro-Therapeutics, Medical and Surgical. A Hand-book for Physicians in the treatment of Nervous and other Diseases.* By Allan McLane Hamilton, M. D. New York: D. Appleton & Co., 1873. Buffalo: Martin Taylor.

The medical profession have been favored lately with a profusion of hand-books upon the application of Electricity to Medicine. That much has been accomplished by many of them, save to keep the subject before the minds of the profession is to be doubted.

A few have however, been received with favor—have really been an aid to practitioners in studying this most interesting, and for the time, popular branch of therapeutics Among those which may be studied with benefit by those desiring information upon the subject is the one under consideration; although imperfect in certain particulars, and hasty and unsupported in some of the conclusions arrived at, as a whole it is an excellent book. The first portion considers Electro-physics and gives a description of the electrical instruments in general use. The author gives preference to those manufactured by the Galvano-Faradic Company of New York, several of whose instruments, are figured. Part Second is a consideration of Electro-Physiology, part third Electro-Therapeutics, and part fourth Special Electro-Therapeutics, the reports of cases are numerous and form an interesting portion of the book

The directions for the application of electricity are well given, and a wise discrimination is exercised in indicating the cases which will be benefitted by its use. Dr. Hamilton has had a large experience in the use of electricity, and is therefore qualified to speak with some authority; we commend his book to those in search of an instructive and interesting guide.

